# The Only Cure

**Published:** 1913-03

**Authors:** Ella Wheeler Wilcox


### The Only Cure.

You may talk of reformations of the Economic Plan
That shall stem the Social Evil in its Course;
But the Ancient Sin of nations must be got at in the Man.
If you want to cleanse a river, seek the source.

Ever since his first beginning, Man has had his way, in lust.
He has never learned the law of Self-Control;
And the World condones his sinning, and the Doctors say he must.
And the Churches shut their eyes, and take his toll.

And the lauded "Lovely Mothers" send the son out into life
With no knowledge-welded armor for the fight;
"He will make his way like others, thro' the Oatfield, to the Wife."
"He will somehow be led onward, to the light."

Yes, his leaders, they shall find him on the highways at each turn
(Since you did not choose to counsel or to warn);
They shall tempt him, then shall bind him; they shall blight, and they shall burn,
Down to offspring and descendants yet unborn.

It can never end through preaching; it can never end through laws;
This social sore no punishment can heal.
It must be the mother's teaching of the purpose and the cause,
And God's glory, lying under sex appeal.

She must feel no fear to name it to the children it has brought;
She must speak of it as sacred, and sublime;
She must beautify, not shame it, by her speech and by her thought,
Till they listen, and respect it, for all time.

From the heart they rested under ere they saw the light of day.
Must the daughters and the sons be taught the truth,
Till they think of it with wonder, as a holy thing alway,
While love's wisdom guides them safely through their youth.

Oh, the world has made its devil, and the Mothers let it grow;
And the Man has dragged their thoughts down to the earth.
There will be no Social Evil when each making mind shall know
All the grandeur and the beauty hid in birth.

When each Mother sets the fashion to win confidence and trust,
And to teach the mighty lesson, Self-Control,
We can lift the great Sex problem from the darkness and the dust,
And enshrine it on the altar of the soul.

—*Ella Wheeler Wilcox.*

